# The NLRP3 Inflammasome Across the AKI–AKD–CKD Continuum: Mitochondrial, Epigenetic and Endocrine–Metabolic Regulation

**DOI:** 10.3390/ijms27209176

**Published:** 2026-10-16

**Authors:** Daniele La Russa, Sabrina Bossio, Antonella La Russa, Michele Di Dio, Daniela Pellegrino, Anna Perri

**Affiliations:** 1 Department of Biology, Ecology and Earth Sciences, University of Calabria, 87036 Rende, Italy; danielapellegrino@unical.it; 2 Department of Pharmacy, Health and Nutritional Sciences, University of Calabria, 87036 Rende, Italy; sabrinabossio1@gmail.com (S.B.); michele.didio@unical.it (M.D.D.); anna.perri@unical.it (A.P.); 3 Department of Health Sciences, Magna Graecia University, 88100 Catanzaro, Italy; lrantonella@yahoo.it; 4 Department of Surgery, Division of Urology, Annunziata Hospital, 87100 Cosenza, Italy; 5 Endocrine and Metabolic diseases Unit, Annunziata Hospital, 87100 Cosenza, Italy

**Keywords:** oxidative stress, chronic kidney disease, renal inflammtion, NLRP3 inflammasome, acute kidney injury

## Abstract

The NLRP3 inflammasome has emerged as a potential molecular link between cellular stress, sterile inflammation, and progressive kidney damage. This review critically examines canonical, non-canonical, and mitochondrial mechanisms of NLRP3 regulation across the acute kidney injury (AKI)–acute kidney disease (AKD)–chronic kidney disease (CKD) continuum, integrating their relationships with pyroptosis, maladaptive repair, fibrosis, and epigenetic regulation. Particular attention is given to endocrine–metabolic signals that may shape renal inflammasome activity. Hyperglycemia, advanced glycation end products, mitochondrial reactive oxygen species, TXNIP signaling, lipotoxicity, impaired tubular fatty-acid oxidation, podocyte insulin resistance, obesity-related adipokine imbalance, and aldosterone-mineralocorticoid receptor activation may converge on NLRP3. Conversely, inflammasome-dependent cytokine release and pyroptosis may amplify insulin resistance, metabolic dysfunction, and cardiorenal injury, establishing a bidirectional endocrine-renal inflammatory loop. Importantly, increased NLRP3 mRNA or protein abundance is distinguished from functional inflammasome activation, which is more strongly supported by downstream evidence such as ASC recruitment/oligomerization, caspase-1 activation, mature IL-1β/IL-18 production, GSDMD cleavage, or pyroptotic cell death. A central issue is whether NLRP3 is a causal driver of kidney disease or a marker of ongoing tissue injury. Although genetic and pharmacological studies support a pathogenic role in experimental models, clinical evidence remains largely associative and disease-dependent. NLRP3-related signals should therefore be regarded as candidate, rather than validated, biomarkers. SGLT2 inhibitors, GLP-1 receptor agonists and finerenone may indirectly attenuate NLRP3 signaling, but mechanistic mediation of their clinical benefits remains unproven. Defining cell-specific activation and clinical target engagement will be essential to establish NLRP3 as a biomarker and therapeutic target.

## 1. Introduction

Acute kidney injury (AKI), acute kidney disease (AKD), and chronic kidney disease (CKD) represent clinically distinct but pathophysiologically interconnected stages of kidney injury and repair [1,2]. AKI is defined by an abrupt decline in kidney function developing within hours to 7 days. AKD encompasses persistent structural or functional kidney abnormalities occurring after an acute insult and lasting from 7 to 90 days, thereby representing an important intermediate phase in which recovery may remain incomplete or maladaptive. CKD requires kidney structural or functional abnormalities persisting for more than 3 months [2]. Thus, the commonly used term “AKI-to-CKD transition” should be understood as a dynamic AKI–AKD–CKD continuum rather than as a direct and uniform progression from acute to chronic disease. Inflammation is a coordinated host response to infection or tissue injury and is a major contributor to this continuum [3,4].

Oxidative stress, regulated cell death, immune-cell recruitment, epithelial phenotypic changes, and extracellular matrix deposition act together during CKD progression. The kidney is also an important target of systemic metabolic and hormonal disturbances. Hyperglycemia, insulin resistance, obesity-related adipokine imbalance, lipotoxicity, and inappropriate aldosterone-mineralocorticoid receptor activation may promote mitochondrial dysfunction, oxidative stress, and sterile inflammation. These signals can converge on the NLRP3 inflammasome in renal immune cells, tubular epithelial cells, and podocytes [5,6,7,8]. Conversely, sustained NLRP3 activation and the release of IL-1β and IL-18 may further contribute to metabolic dysfunction, systemic inflammation, and cardiorenal injury. Epigenetic mechanisms may additionally sustain inflammatory and profibrotic responses after the initial metabolic insult, contributing to the concept of metabolic memory of diabetic kidney disease [9].

Within this complex network, NLRP3 may represent an important molecular interface linking cellular stress, inflammation, mitochondrial dysfunction, and metabolic disturbances across the AKI–AKD–CKD continuum. However, its biological role is likely to vary with disease stage: acute inflammasome signaling may amplify injury during AKI, persistent signaling may contribute to incomplete recovery and maladaptive repair during AKD, whereas chronic inflammatory and fibrotic circuits may predominate in established CKD. The extent to which NLRP3 acts as a causal driver of renal disease, rather than as a marker or amplifier of ongoing tissue injury, remains incompletely defined. In this review, we discuss canonical and non-canonical pathways of NLRP3 activation in kidney disease, with particular emphasis on mitochondrial and epigenetic regulation and on endocrine–metabolic signals that may modulate inflammasome activity. We further examine the potential role of NLRP3 as a biomarker and therapeutic target, highlighting the gap between experimental evidence and clinical validation.

## 2. NLRP3 Inflammasome: Structure and Activation

Inflammasomes are macromolecular signaling complexes of the innate immune system that respond to danger signals and promote caspase-1 activation, maturation of interleukin-1β (IL-1β) and IL-18, and, under appropriate conditions, gasdermin D (GSDMD)-dependent pyroptosis [10]. Inflammasome-forming sensors include members of the nucleotide-binding domain-like receptor (NLR) family, such as NLRP3 and NLRC4, and non-NLR sensors, such as absent in melanoma 2 (AIM2) and pyrin [11,12,13,14].

Toll-like receptors (TLRs), although not inflammasome receptors, can provide a transcriptional priming signal through pathways including MyD88/NF-κB. The core inflammasome consists of a sensor, an adaptor, and an effector protease. In the NLRP3 inflammasome, the sensor NLRP3 recruits apoptosis-associated speck-like protein containing a CARD (ASC), which in turn recruits pro-caspase-1, enabling its activation and downstream cytokine processing. Pathogen-associated molecular patterns (PAMPs) and damage-associated molecular patterns (DAMPs) can initiate these responses, with DAMPs released during ischemia, metabolic stress, and tissue injury contributing to sterile inflammation [15,16,17,18]. NLRP3 is the most extensively studied inflammasome sensor because it responds to a broad range of cellular disturbances, including extracellular ATP, ionic fluxes, crystals, mitochondrial dysfunction, and microbial products [19]. The NLRP3 protein structure mainly includes a C-terminal leucine-rich (LRR), central nucleotide-binding oligomerization domain (NACHT), and N-terminal pyrin domain (PYD) [18,20]. Following activation, NLRP3 oligomerization promotes ASC recruitment and assembly of the inflammasome complex, leading to pro-caspase-1 activation. Activated caspase-1 induces IL-1β and IL-18 maturation and can trigger GSDMD-dependent pyroptosis, characterized by plasma membrane pore formation, secretion of inflammatory mediators, DNA damage, and osmotic disintegration of cells [11]. These mechanisms provide the molecular basis for the role of NLRP3 in renal inflammation and tissue injury (Table 1).

### 2.1. NLRP3 Signal Pathways: New Insights in Canonical Activation in Renal Diseases

Canonical NLRP3 inflammasome activation is commonly described using a two-signal framework comprising priming and activation; however, this model is a useful conceptual simplification rather than a strictly sequential and universally sufficient mechanism. NLRP3 activity is controlled by multiple regulatory layers, including transcriptional priming, post-transcriptional regulation, post-translational modifications such as phosphorylation and ubiquitination/deubiquitination, subcellular trafficking, ionic fluxes, organelle stress and interactions, metabolic signals, and NEK7-dependent inflammasome assembly. Priming increases the expression of inflammatory precursors, including pro-IL-1β and NLRP3, mainly through pattern-recognition receptors and inflammatory signaling pathways such as TLR4/MyD88/NF-κB [21,22,23,24]. TLR4 is one of the best-characterized TLRs in AKI, and its expression under physiological conditions is widely distributed in renal parenchymal cells and resident immune cells [25]. In the renal cortex, TLR4 is detectable in proximal and distal tubules, as well as in podocytes, glomerular mesangial cells, peritubular endothelial cells, and collecting duct cells [26]. Under normal conditions, renal TLR4 expression is low; however, its expression is enhanced in response to renal injury and/or infection [27]. TLR4 signaling therefore represents an important priming mechanism linking renal injury to increased NLRP3 pathway competence, but priming alone does not establish functional inflammasome activation. In addition to transcriptional regulation, NLRP3 expression and activation competence can be modulated by post-transcriptional and post-translational mechanisms, including non-coding RNAs, deubiquitination and phosphorylation [28,29,30].

Several miRNAs, including miR-21-5p, miR-125a-5p, miR-10a/b, miR-155 and miR-30c, have been reported to modulate TLR4/NF-κB/NLRP3 signaling in experimental models of AKI and CKD, although their effects appear to be context-dependent [31,32,33,34]. Increased NLRP3 mRNA or protein abundance should be interpreted as pathway upregulation or priming and not, by itself, as evidence of functional inflammasome activation. In the conventional framework, an additional activation signal generated by cellular stress and damage can promote NLRP3 assembly; such signals include potassium efflux, mitochondrial perturbation, reactive oxygen species (ROS), lysosomal damage, and other DAMP-associated events. Functional activation is more convincingly demonstrated by downstream readouts such as ASC recruitment or oligomerization, caspase-1 cleavage or enzymatic activity, maturation/release of IL-1β and IL-18, GSDMD cleavage, and pyroptotic cell death. Zheng et al. demonstrated that oxidative stress induces alternative polyadenylation (APA) by causing trans-factor FIP1 to bind to the proximal PAS of the NLRP3 3′UTR via its arginine-rich domain and induces NLRP3 3′UTR shortening. Such 3′UTR shortening amplifies inflammation, fibrogenesis, ROS production, and apoptosis in kidney tubular epithelial cells by enhancing mRNA stability and NLRP3 protein expression [35]. This study therefore primarily supports regulation of NLRP3 abundance rather than, on that basis alone, definitive inflammasome activation. In early CKD models, increasing uric acid was associated with upregulation of IL-1β and ICAM-1 together with ROS generation and K+ efflux; where downstream inflammasome readouts were measured, these findings support functional NLRP3 activation [36].

Overall, the available evidence supports a role for canonical NLRP3 signaling in renal inflammation and injury, but its regulation is highly context-dependent, and the translational relevance of many proposed mechanisms remains uncertain. Importantly, changes in NLRP3 abundance alone should not be considered sufficient evidence of inflammasome activation. Stronger evidence requires complementary readouts, including ASC oligomerization or speck formation, caspase-1 activation, mature IL-1β/IL-18 release, and GSDMD cleavage. Moreover, although pharmacological and genetic modulation of NLRP3-related pathways can reduce renal injury in experimental models, these findings do not establish that NLRP3 modulation mediates clinical renoprotection in patients.

### 2.2. Non-Canonical and Mitochondrial NLRP3 Activation in Renal Diseases

Non-canonical inflammasome signaling is initiated by cytosolic lipopolysaccharide (LPS), which directly activates caspase-4 or caspase-5 in humans and caspase-11 in mice [37]. This initial step is independent of caspase-1 and results in GSDMD cleavage and membrane-pore formation. The resulting potassium efflux can subsequently activate the canonical NLRP3-ASC-caspase-1 complex, promoting maturation of IL-1β and IL-18. Experimental evidence suggests that this pathway may also contribute to renal injury. In ischemia/reperfusion injury, increased caspase-11 expression and pannexin 1 (panx1)-cleavage were associated with NLRP3 inflammasome activation, caspase-1 activation, and IL-1β maturation [38].

Mitochondrial dysfunction and mitochondrial reactive oxygen species (mtROS) are important, but context-dependent, upstream signals that can facilitate NLRP3 inflammasome activation rather than universal or obligatory mediators [39]. Mitochondrial stress can influence NLRP3 through several interconnected processes, including altered redox signaling, mitochondrial DNA release, changes in membrane potential, impaired mitophagy, metabolic reprogramming, and organelle crosstalk. In renal tubular cells, hypoxia has been associated with increased NLRP3 expression and mitochondrial redistribution accompanied by mitochondrial oxidative stress changes [40]; because this study also describes inflammasome-independent functions of NLRP3, increased expression should not be equated automatically with assembly of the canonical ASC–caspase-1 complex. In diabetic nephropathy, CD36-mediated impairment of mitochondrial fatty-acid oxidation promotes mtROS production and has been linked to NLRP3 inflammasome activation in renal tubular epithelial cells, while thioredoxin and thioredoxin-interacting protein (TXNIP)/NLRP3/IL-1β signaling contribute to oxidative tubular injury [5,41]. These observations support mtROS as one component of a broader mitochondrial danger-signaling network whose contribution varies according to cell type, metabolic state, disease stage, and experimental model.

Conversely, preservation of mitochondrial quality control may limit inflammasome activation. Prohibitin 2, a newly identified intracellular receptor of mitophagy, protected NLRP3-induced inflammatory pathways through amelioration of mitochondrial dysfunction in renal tubular cells [42], while Peroxisome proliferator-activated receptor-gamma coactivator (PGC)-1 alpha induction improved mitochondrial dynamics and morphology and attenuated oxidative stress levels and NLRP3 inflammasome activation in primary renal tubular epithelial cells [43]. In renal fibrosis, the HIF1α-BNIP3 signaling pathway is involved in mitophagy and plays a protective role by decreasing mitochondrial ROS and inhibiting NLRP3 inflammasome activation [44]. Crosstalk between mitochondria and the endoplasmic reticulum may provide an additional regulatory mechanism: in renal ischemia/reperfusion injury (IRI), IRE1α-XBP1 signaling was reported to promote NLRP3 expression and downstream inflammatory responses [45]. In patients with progressive glomerulosclerosis, Silent mating type information regulation 2 homolog 1 (SIRT1) was significantly decreased in the glomeruli, and in cultured podocytes, overexpression of SIRT1 inhibited NLRP3 activation, protected against mitochondrial dysfunction, and protected podocytes from aldosterone-induced injury [46]. Administration of αKlotho protein protected the kidney and HK-2 cells against contrast-induced injury by promoting autophagy via inhibition of the AKT/mTOR pathway and decreasing mitochondrial ROS levels [47]. Several pieces of evidence suggest that free mtDNA is a significant biomarker during the occurrence and progression of AKI, but it can also serve as a DAMP to trigger the innate immune system to initiate a non-infection-related inflammatory response and further mediate tubular cell inflammation and apoptosis [48]. In this scenario, mitochondria are becoming a new focus for CKD therapy, and recently miR-214 was identified as an endogenous mitochondrial regulator in renal tubular cells and a common target for renal tubular protection in different CKD conditions. Pharmacologic inhibition of miR-214 attenuated apoptosis, inflammation, fibrosis, and mitochondrial damage in these CKD models [49].

Mitochondrial-derived signals may therefore participate in a reciprocal relationship with NLRP3 signaling. mtROS, oxidized mitochondrial molecules, and cytosolic mtDNA can facilitate inflammasome activation, whereas effective mitophagy may limit the availability of these activating signals. Recent analyses identify the mitophagy-NLRP3 balance as a candidate determinant of maladaptive repair and AKI-to-CKD progression [50]. Experimental evidence also implicates ISG15 as an upstream promoter of tubular-cell pyroptosis through an mtDNA/STING-related pathway converging on NLRP3-CASP1-GSDMD in diabetic kidney disease [51]. Most data in this area derive from cultured tubular cells, genetically or pharmacologically manipulated rodents, and disease-specific experimental models. They provide stronger evidence for a driver role when inhibition or deletion of a pathway component reduces injury, but extrapolation to human CKD remains limited by model dependence, differences in disease stage, and the absence of serial kidney-tissue measurements in patients. Clinical observations, such as reduced glomerular SIRT1 in progressive glomerulosclerosis, are associative and cannot by themselves establish that NLRP3 activation caused progression. In addition, mitochondrial localization and inflammasome-independent functions of NLRP3 should be distinguished from formation of the canonical ASC-caspase-1 complex (Table 1).

**Table 1:** Overview of NLRP3 inflammasome activation mechanisms in kidney disease. The table summarizes canonical, non-canonical, mitochondrial, and mitophagy-related mechanisms implicated in renal NLRP3 activation, including major triggers, molecular pathways, renal disease contexts, downstream effects, and the overall level of experimental or human evidence. Canonical activation involves TLR4/NF-κB-dependent priming followed by NLRP3–ASC–caspase-1 activation, whereas non-canonical signaling is initiated by cytosolic LPS-dependent caspase-4/5 activation in humans or caspase-11 activation in mice. Mitochondrial mechanisms include mtROS, mtDNA, and impaired mitochondrial homeostasis, while mitophagy-related pathways may modulate NLRP3 activity through regulators of mitochondrial quality control.

Mechanism	Main Triggers/Signals	Molecular Pathway	Renal Context	Main Downstream Effects	Evidence Category	References
Canonical	ATP, K^+^ efflux, ROS, DAMPs	TLR4/NF-κB → NLRP3 → ASC → caspase-1	AKI, CKD, diabetic kidney disease	IL-1β, IL-18, pyroptosis	Animal models + cell culture	[25,26,27,36,52]
Non-canonical	Cytosolic LPS	Caspase-4/5 (human), caspase-11 (mouse) → GSDMD → K^+^ efflux → NLRP3	IRI/AKI	GSDMD cleavage, IL-1β/IL-18	Animal renal model + indirect mechanistic evidence	[37,38]
Mitochondrial	mtROS, mtDNA, mitochondrial dysfunction	mtROS/TXNIP → NLRP3	Tubular injury, DKD, fibrosis	Inflammasome activation, pyroptosis	Animal models + cell culture	[5,40,41]
Mitophagy-related	Impaired mitochondrial quality control	PGC-1α, PHB2, BNIP3, etc.	CKD/fibrosis	Reduced/enhanced NLRP3 activation	Animal models + cell culture	[42,43,44,45]

## 3. Epigenetic and Genetic Regulation of NLRP3 Inflammasome in CKD

Epigenetic mechanisms regulate gene expression without altering the underlying DNA sequence and include DNA and RNA methylation, histone modifications, and non-coding RNAs such as microRNAs (miRNAs), long non-coding RNAs (lncRNAs) and circular RNAs (circRNAs) [53].

A growing body of evidence has demonstrated that genetics and epigenetics are deeper mechanisms regulating renal fibrosis, the common outcome across all kinds of progressive CKD. The pathogenetic mechanisms leading to CKD are very complicated and not completely elucidated yet, because many questions are still open, such as which kind of cells drive fibrosis and how cells communicate with each other [8]. In this scenario, many pro-fibrotic conditions could control genome epigenetic reprogramming, further contributing to the progression of kidney disease [9].

Therefore, the reversible nature of epigenetic changes could represent a useful tool to counteract the onset of fibrosis or slow its progression. Importantly, the evidence base is heterogeneous and includes both renal disease models and mechanistic studies performed outside kidney disease. In renal experimental models, miR-377 has been associated with oxidative stress and fibrosis through the p38 MAPK/TXNIP/NLRP3 pathway in diabetic nephropathy, while miR-155 has been reported to promote NLRP3-related signaling in hypoxic tubular cells by targeting FOXO3a [54,55]. By contrast, several observations concerning promoter methylation and histone acetylation derive from non-renal or only indirectly renal experimental contexts. *Mycobacterium tuberculosis*-induced demethylation of the NLRP3 promoter was demonstrated in human embryonic kidney-derived cells, an infection model that provides mechanistic evidence for epigenetic regulation but not direct evidence of NLRP3-mediated renal pathology [56]. A case–control study in patients with type 2 diabetes associated lower NLRP3 promoter methylation with diabetic microvascular complications; this human evidence is observational and is not specific to kidney tissue [57]. Likewise, low-dose epirubicin reduced histone H3 lysine 9 acetylation and downregulated NLRP3 inflammasome components in primary macrophages [58], whereas bortezomib-induced painful neuropathy was associated with STAT3-dependent histone H3/H4 acetylation at the NLRP3 promoter in a non-renal disease model [59]. These findings should therefore be considered mechanistic or hypothesis-generating for kidney disease rather than direct renal evidence. More directly relevant human renal evidence is provided by altered NLRP3 promoter methylation reported in adult and pediatric patients with idiopathic nephrotic syndrome and glucocorticoid resistance [60]. Overall, epigenetic association should not be equated with causal regulation of CKD progression. Before epigenetic therapies can be considered safe and effective approaches to attenuate NLRP3-mediated renal inflammation, findings require replication in complementary preclinical systems, validation in human kidney tissue, and prospective clinical studies.

During the last decades, genetic variation may represent an additional layer of NLRP3 regulation. Functional studies revealed that some of these SNPs may change NLRP3 function, increasing (gain-of-function) or decreasing (loss-of-function) the activation of NLRP3 machinery [61]. Despite the consolidated and well-recognized role of NLRP3 inflammasome dysregulation in CKD development, few efforts have been directed toward the research of the potential pathogenetic role of NLRP3 inflammasome polymorphisms in CKD. To date, some functional variants of NLRP3 and its components have been found to be associated with an increased risk of lupus nephritis, diabetic nephropathy, and CKD, including variants in NLRP3 and CARD8 [61,62,63,64]. However, these associations remain exploratory and require replication in larger, independent, and genetically diverse cohorts, together with functional validation in disease-relevant renal and immune cells. Accordingly, genetic variants should not yet be considered established tools for predicting CKD progression. More recent studies have extended the concept of epigenetic regulation to extracellular vesicle-mediated transfer of regulatory RNAs. Exosome-associated miR-223-3p and circDENND4C have been reported to attenuate NLRP3-dependent pyroptosis in an experimental model of acute kidney injury [65,66]. These findings support the possibility of intercellular transfer of regulatory RNAs but remain preclinical. Overall, the available evidence indicates that epigenetic and genetic mechanisms can modulate NLRP3 signaling, while their contribution to human CKD remains incompletely defined. Observational and cross-sectional studies cannot establish causality, and epigenetic profiles measured in circulating cells may not reflect those of specific renal cell populations. Future studies should therefore integrate cell-specific epigenomic analyses with functional validation and longitudinal clinical phenotyping (Table 2).

## 4. Stage-Specific Roles of NLRP3 Across AKI, AKD and CKD: Implications for Biomarker Development

**Figure 1:**
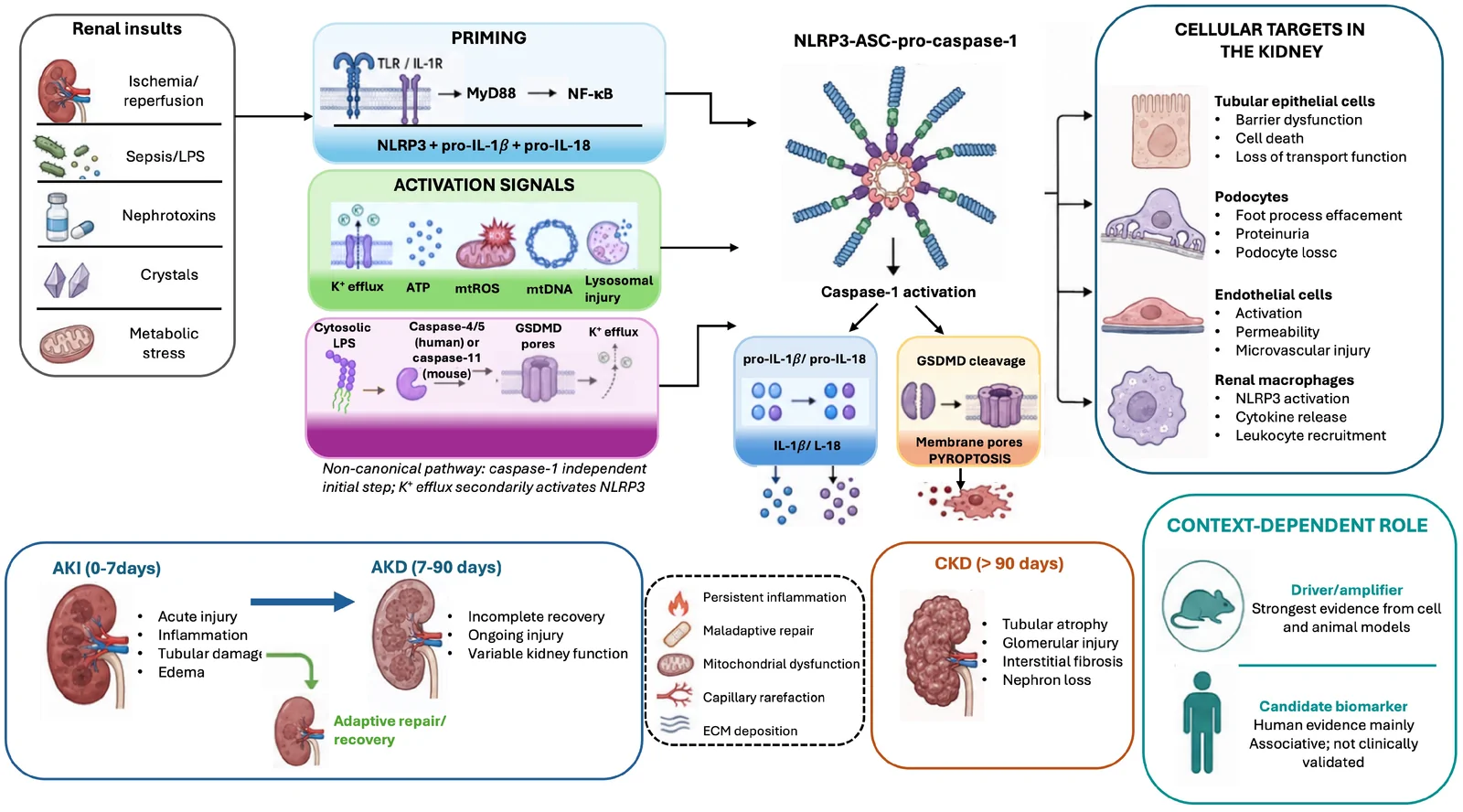
NLRP3 Inflammasome Activation across the AKI–AKD–CKD Continuum. Renal insults provide priming and activation signals that promote NLRP3 inflammasome assembly, caspase-1 activation, IL-1β/IL-18 maturation and GSDMD-dependent pyroptosis. While adaptive repair may restore renal function, persistent inflammation, mitochondrial dysfunction, and maladaptive repair may drive AKI progression through AKD to CKD. NLRP3 may act as a context-dependent driver or amplifier of injury, although its clinical value as a biomarker remains unvalidated.

The AKI–AKD–CKD continuum comprises biologically distinct phases rather than a single homogeneous process (Figure 1). During AKI (up to 7 days), abrupt tubular, endothelial, and immune-cell stress generates DAMPs, ionic disturbances, mitochondrial dysfunction, and other signals that can promote NLRP3 inflammasome assembly and amplify acute inflammatory injury. During AKD (7–90 days), incomplete recovery may be characterized by persistent immune-cell recruitment, unresolved tubular stress, altered mitochondrial quality control, cell-cycle dysregulation, fibroblast activation, and maladaptive extracellular-matrix deposition; in this phase, sustained NLRP3-related signaling may function predominantly as an amplifier of maladaptive repair [67,68]. In established CKD (>90 days), the biological context shifts toward chronic inflammation, tubular atrophy, interstitial fibrosis, capillary rarefaction, and metabolic stress, in which NLRP3 may contribute to maintenance or progression of injury but may also simply mark ongoing tissue damage. Experimental ischemia–reperfusion studies have associated persistent NLRP3 expression with macrophage infiltration, tubular lesions, and fibrosis [35]. Because persistent expression alone does not demonstrate active inflammasome assembly, such findings should be interpreted separately from studies documenting downstream events such as ASC recruitment, caspase-1 activation, IL-1β/IL-18 maturation, GSDMD cleavage, or pyroptotic cell death. These stage-specific distinctions are important when considering NLRP3 as a biomarker of AKI-to-CKD progression. Clinical validation would require standardized assays, prospective human cohorts, serial sampling or non-invasive surrogate measurements, and demonstration of incremental prognostic value beyond eGFR, albuminuria, and established clinical variables. The pathophysiology of ischemic AKI is also associated with endothelial and epithelial cell injury [69], and administration of endothelial progenitor cells exerts a renoprotective effect in an IRI mouse model through down-regulation of NLRP3, cleaved caspase-1, p-NF-κB protein levels, and reduction in epithelial–mesenchymal transition (EMT)-associated factors [70]. Deposition of extracellular matrix, EMT, and inflammation are crucial processes in CKD progression, and matrix metalloproteinases (MMPs) are involved in ECM degradation [71]. Among the MMP family, MMP7 also regulates non-ECM processes such as cell proliferation, EMT, and apoptosis [72,73], and its mRNA level was found to be markedly upregulated in kidney tissues of CKD patients. In kidney cells, MMP7 overexpression increased NLRP3/NLRP6-related inflammasome signaling and fibrosis-related markers, while experimental MMP7 inhibition attenuated these effects [52]. Persistent inflammation followed by renal fibrosis and sustained activation of the renin-angiotensin system, NF-κB system, and NLRP3/IL-1β pathway were observed in gentamicin-induced AKI, suggesting that targeting these pathways may represent an experimental strategy to limit progression [74]. A prospective cohort study assessed 3123 participants with stages 2 to 4 CKD and demonstrated that higher 24 h urinary oxalate excretion may be a risk factor for CKD progression and end-stage renal disease (ESRD) [75]. This clinical association is consistent with experimental studies linking calcium oxalate-induced kidney injury to intrarenal NLRP3 inflammasome activation [76], including indirect activation mediated by ATP released from damaged tubular epithelial cells [77]. Future basic and clinical studies are needed to determine whether NLRP3-related measurements have reproducible predictive value and whether biomarker-defined subgroups identify patients likely to benefit from pathway-directed interventions.

A critical question emerging from the available literature is whether NLRP3 activation represents a causal driver of renal injury and disease progression or primarily reflects ongoing tissue damage as a biomarker. A driver role is most strongly supported in experimental systems in which genetic deletion or pharmacological inhibition of NLRP3-pathway components reduces caspase-1 activation, pyroptosis, inflammation, and subsequent fibrosis. Examples include crystal nephropathy, ischemia–reperfusion injury, diabetic kidney disease, and selected toxic or obstructive models. However, even these experiments may be influenced by developmental effects of germline knockout, off-target drug actions, differences between immune and parenchymal cells, and intervention before or at the time of an acute insult rather than after established CKD. Conversely, most evidence in patients consists of cross-sectional associations between NLRP3 expression, inflammatory infiltrates, fibrosis, or disease severity. Such data are compatible with NLRP3 being activated downstream of tissue damage and functioning as a marker or amplifier rather than the initiating cause. Recent syntheses emphasize that renal macrophages contain the complete NLRP3 machinery and can amplify GSDMD-dependent pyroptosis and fibrogenic signaling, but macrophage phenotype and disease stage may also permit reparative functions [78,79]. Moreover, NLRP3 can exert inflammasome-independent effects in renal epithelial cells. The most defensible interpretation is therefore that NLRP3 acts as a disease-modifying driver in selected molecular and temporal contexts, while in others its abundance primarily reflects ongoing injury. Demonstrating causality in human CKD will require longitudinal studies with validated target-engagement markers and, ultimately, clinical evidence that selective NLRP3 inhibition alters kidney outcomes.

Translationally, direct NLRP3 inhibitors such as MCC950 and newer small molecules, as well as interventions targeting upstream triggers, IL-1beta/IL-18 signaling, or GSDMD-dependent pyroptosis, have shown benefit in experimental kidney models [80,81]. No NLRP3-directed drug is currently approved specifically for CKD. The gap between preclinical efficacy and clinical translation reflects CKD heterogeneity, cell- and stage-specific functions of NLRP3, pharmacokinetic and safety considerations, and the need to preserve physiological host defense. Importantly, the clinical renoprotective efficacy of treatments such as SGLT2 inhibitors should not be presented as proof of NLRP3-dependent benefit merely because these agents reduce inflammasome readouts in experimental models. Future trials should incorporate pharmacodynamic measures of target engagement, prespecified molecular phenotypes and longitudinal kidney outcomes. Kidney-selective delivery and integration of genetic, epigenetic and pyroptosis signatures may help identify the subgroup in which persistent NLRP3 activity is mechanistically linked to progression.

## 5. Endocrine–Metabolic Regulation of the Renal NLRP3 Inflammasome

**Figure 2:**
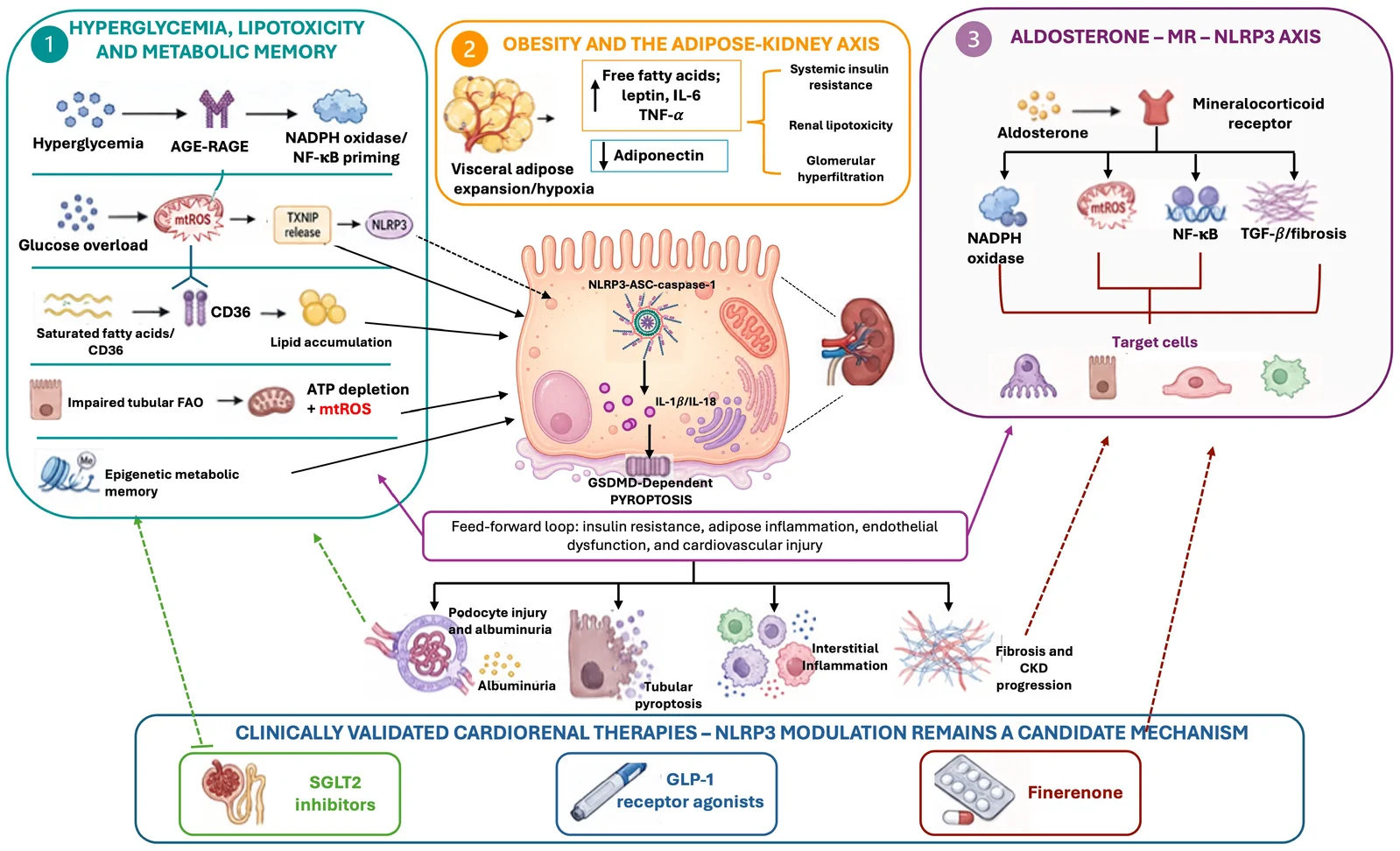
Endocrine–Metabolic Regulation of the Renal NLRP3 Inflammasome. Hyperglycemia, mitochondrial oxidative stress, lipotoxicity, impaired fatty-acid oxidation, obesity-related adipokine imbalance and aldosterone–mineralocorticoid receptor signaling may converge on renal NLRP3 activation. NLRP3-mediated inflammation and pyroptosis may reciprocally aggravate metabolic and kidney dysfunction. SGLT2 inhibitors, GLP-1 receptor agonists and finerenone may attenuate these pathways, although NLRP3-dependent clinical target engagement remains unproven.

The kidney is both a target and a regulator of endocrine and metabolic signals. In chronic kidney disease (CKD), hyperglycemia, insulin resistance, adipose-tissue dysfunction and inappropriate mineralocorticoid receptor activation converge on oxidative stress, mitochondrial dysfunction and sterile inflammation. The NLRP3 inflammasome may integrate these signals in renal immune cells, podocytes and tubular epithelial cells. Conversely, sustained NLRP3 activation and the consequent release of IL-1β and IL-18 may amplify systemic insulin resistance, adipose-tissue inflammation, endothelial dysfunction and cardiovascular injury, thereby contributing to the endocrine–metabolic complications of CKD. However, most direct mechanistic evidence has been obtained in cultured cells and animal models; in patients, the relationship between metabolic abnormalities, NLRP3 activation, and kidney outcomes remains predominantly associative. The principal endocrine–metabolic signals implicated in renal NLRP3 regulation are summarized in Table 3 and illustrated in Figure 2.

Chronic hyperglycemia promotes NLRP3 activation through multiple, partly overlapping mechanisms. Increased glucose flux enhances the generation of advanced glycation end products (AGEs), whose interaction with the receptor for AGEs (RAGE) activates NADPH oxidases, NF-κB, and pro-inflammatory gene transcription. This pathway may provide the priming signal required for increased expression of NLRP3 and pro-IL-1β. At the same time, glucose overload increases mitochondrial electron leakage and mitochondrial reactive oxygen species (mtROS), promoting oxidation and dissociation of thioredoxin-interacting protein (TXNIP) from thioredoxin. Free TXNIP can interact with NLRP3, linking oxidative stress to inflammasome assembly and caspase-1 activation [5,82]. Damaged mitochondria may further amplify this response by releasing mitochondrial DNA and other damage-associated molecular patterns.

Podocytes are particularly vulnerable to this metabolic-inflammatory interaction. Insulin signaling is required to preserve cytoskeletal organization, glucose uptake, and slit-diaphragm integrity. Podocyte insulin resistance may therefore impair cellular energy homeostasis and increase susceptibility to oxidative stress. Hyperglycemia, AGEs, and saturated fatty acids can also induce NLRP3-caspase-1-GSDMD signaling, leading to podocyte pyroptosis, foot-process effacement, and albuminuria. NLRP3 inhibition attenuated podocyte injury and lipid accumulation in experimental diabetic nephropathy, supporting a causal contribution within these models, although comparable target-engagement data are not yet available in patients [83]. A parallel process occurs in tubular epithelial cells, which rely predominantly on mitochondrial fatty-acid oxidation (FAO) to meet their high energy requirements. Suppression of PPARα- and PGC-1α-regulated FAO causes intracellular lipid accumulation, ATP depletion, dedifferentiation, and cell death and has been demonstrated in fibrotic human kidney samples as well as in experimental models [6]. Lipid uptake through CD36 may further increase the intracellular burden of long-chain fatty acids and toxic lipid intermediates, including diacylglycerols and ceramides. These molecules promote endoplasmic reticulum stress, mtROS production, and TXNIP–NLRP3 activation. In diabetic tubular cells, CD36-dependent impairment of FAO has been associated with mitochondrial oxidative stress and NLRP3 activation [41]. Thus, NLRP3 may act both downstream of metabolic dysfunction and as an amplifier of tubular inflammation, pyroptosis, and fibrosis.

Transient improvement in glycemic control may not immediately reverse these pathways. Previous hyperglycemic exposure can induce persistent DNA methylation, histone modifications, and non-coding RNA profiles that maintain oxidative, inflammatory, and profibrotic gene expression, a phenomenon defined as metabolic memory [9]. Persistent epigenetic regulation of TXNIP and NF-κB-related pathways provides a plausible link between previous glucose exposure and prolonged inflammasome susceptibility. Nevertheless, direct evidence that NLRP3 is indispensable for metabolic memory in human diabetic kidney disease remains limited. It is more appropriate to consider NLRP3 as one component of an epigenetically maintained inflammatory network rather than its only molecular executor.

Obesity provides a second endocrine–metabolic environment favorable to NLRP3 activation. Expansion and hypoxia of visceral adipose tissue promote adipocyte stress, lipolysis, and macrophage recruitment, increasing circulating free fatty acids, leptin, TNF-α, and IL-6 while reducing the protective adipokine adiponectin. Saturated fatty acids may provide both NF-κB-dependent priming and mitochondrial or lysosomal signals for inflammasome activation. The resulting adipose inflammation contributes to systemic insulin resistance and increases the delivery of glucose and lipids to the kidney. At the renal level, hyperfiltration and glomerular hypertension coexist with ectopic lipid accumulation and inflammatory signaling. CD36-mediated lipid uptake has been linked to podocyte NLRP3 activation and injury in experimental obesity-related glomerulopathy [84]. Conversely, adiponectin may protect podocytes by improving fatty-acid handling, activating AMPK and limiting oxidative stress. In experimental obesity-related glomerulopathy, adiponectin deficiency increased ROS production and NLRP3 activation, whereas restoration of adiponectin signaling attenuated glomerular injury [85]. These observations support an adipose-kidney axis in which an altered leptin/adiponectin balance, lipotoxicity, and insulin resistance converge on renal NLRP3. However, these mechanistic relationships remain mainly preclinical, and circulating adipokines are influenced by reduced renal clearance in CKD, complicating causal interpretation in patients. The interaction is potentially bidirectional. NLRP3-dependent IL-1β release can interfere with insulin-receptor signaling and promote adipose-tissue macrophage activation, while IL-18 participates in complex, context-dependent regulation of energy homeostasis. Progressive CKD may therefore establish a feed-forward loop involving uremic inflammation, adipokine dysregulation, insulin resistance, and further kidney injury. The relative contribution of renal versus adipose or myeloid NLRP3 to this systemic phenotype has not yet been established.

Inappropriate activation of the aldosterone-mineralocorticoid receptor (MR) pathway provides a direct endocrine connection with renal NLRP3 signaling. Beyond its effects on sodium retention and blood pressure, MR activation in podocytes, tubular epithelial cells, endothelial cells, macrophages, and fibroblasts promotes NADPH oxidase activity, mitochondrial dysfunction, inflammation, and extracellular matrix deposition. Aldosterone-induced mtROS production has been shown to activate the NLRP3-caspase-1-IL-1β pathway in cultured tubular cells and experimental animals, whereas mitochondrial antioxidant treatment or NLRP3 inhibition attenuated injury [7]. SIRT1 overexpression similarly reduced mitochondrial dysfunction and NLRP3 activation in aldosterone-exposed podocytes [46]. MR activation can also promote GSDMD-dependent pyroptosis. Eplerenone reduced NLRP3, caspase-1, and pyroptotic cell-death markers in experimental renal injury, supporting the concept that MR blockade may interfere with the inflammasome pathway [86]. Nevertheless, aldosterone activates several parallel profibrotic pathways, including SGK1, Rac1, NF-κB, and TGF-β/Smad signaling. NLRP3 should therefore be viewed as a plausible mediator and amplifier of MR-dependent injury rather than its exclusive downstream mechanism.

## 6. Therapeutic Modulation of NLRP3 by SGLT2 Inhibitors, GLP-1 Receptor Agonists and Finerenone

SGLT2 inhibitors, glucagon-like peptide-1 receptor agonists (GLP-1RAs) and finerenone provide clinically relevant cardiorenal therapies that may also modulate the metabolic and inflammatory pathways associated with NLRP3 activation. SGLT2 inhibitors reduce proximal tubular glucose and sodium reabsorption, and may attenuate glucotoxicity, mtROS, lipotoxicity and improve autophagy and mitochondrial quality control. In experimental CKD, SGLT2 inhibition reduced NLRP3 activation, while dapagliflozin attenuated podocyte pyroptosis through a proposed miR-155-5p/HO-1/NLRP3 pathway [87,88]. However, these mechanisms remain predominantly preclinical, and the established renoprotective effects of SGLT2 inhibitors cannot.

GLP-1RAs may similarly influence NLRP3 signaling through both metabolic and direct anti-inflammatory mechanisms, including improved glycemic control, reduced lipotoxicity, and adipose inflammation, and modulation of cAMP/PKA, AMPK, SIRT1, and Nrf2 signaling. These pathways can improve mitochondrial function and autophagy while suppressing NF-κB priming and mtROS–TXNIP–NLRP3 activation. Experimental studies have linked GLP-1RA treatment to reduced NLRP3-mediated podocyte pyroptosis [89]. Moreover, in patients with type 2 diabetes and CKD, semaglutide reduced major kidney outcomes in the FLOW trial [90]; however, the trial did not assess NLRP3 activity and therefore does not establish NLRP3-dependent target engagement. Finerenone, a selective non-steroidal mineralocorticoid receptor (MR) antagonist, reduces kidney and cardiovascular events in patients with type 2 diabetes and CKD [91,92]. By inhibiting MR-dependent transcription, finerenone may reduce oxidative stress, macrophage activation, inflammatory signaling, and fibrosis and could consequently decrease NLRP3 priming and activation. Nevertheless, clinical trials have not directly assessed renal NLRP3 activity. Overall, these therapies should be regarded as clinically validated cardiorenal therapies that may indirectly and, according to preclinical evidence, partly directly attenuate NLRP3 signaling. Demonstrating this mechanism in patients will require validated circulating or urinary target-engagement biomarkers and longitudinal studies linking changes in inflammasome activity to kidney outcomes (Table 3).

**Table 3:** Endocrine–metabolic regulation and therapeutic modulation of renal NLRP3 signaling. The table summarizes major metabolic and endocrine factors that may promote renal NLRP3 activation, their proposed molecular mechanisms, principal renal cellular targets, and potential therapeutic strategies. Hyperglycemia, lipotoxicity, obesity-related adipokine imbalance, and aldosterone–mineralocorticoid receptor activation are presented as upstream metabolic or endocrine signals, whereas SGLT2 inhibitors, GLP-1 receptor agonists, and finerenone are shown as clinically established cardiorenal therapies that may indirectly modulate NLRP3-related pathways. The “Evidence in humans” column highlights the distinction between established clinical benefits and the still-unproven role of NLRP3-dependent target engagement.

Factor	Effect on Renal NLRP3	Main Mechanism	Renal Target/Cell	Therapeutic Strategy	Evidence Setting	Human/NLRP3 Evidence	References
Hyperglycemia	↑	AGE/RAGE, NF-κB, mtROS, TXNIP	Tubular cells, podocytes	Glycemic control	in vitro/ in vivo	Indirect	[5,82]
Lipotoxicity	↑	CD36, impaired FAO, mtROS	Tubular cells, podocytes	Metabolic modulation	in vitro/ in vivo/ human tissue	Limited	[5,6,41,93,94]
Obesity/adipokine imbalance	↑	FFA, leptin/adiponectin imbalance, ROS	Podocytes/adipose-kidney axis	Weight/metabolic control	in vivo	Associative	[84,85]
Aldosterone/MR activation	↑	NADPH oxidase, mtROS, NF-κB	Tubules, podocytes, macrophages	MR antagonism	in vitro/ in vivo	Clinical MR benefit; NLRP3 not assessed	[7,46,86]
SGLT2 inhibitors	↓?	Glucotoxicity, mtROS, autophagy	Tubular/podocyte	Dapagliflozin etc.	in vivo	NLRP3 target engagement unproven	[87,88]
GLP-1RAs	↓?	AMPK/SIRT1/Nrf2, reduced lipotoxicity	Podocytes/metabolic axis	Semaglutide etc.	in vivo/human clinical	Clinical benefit demonstrated; NLRP3 not assessed	[89,90]
Finerenone	↓?	MR inhibition, reduced oxidative/inflammatory signaling	Multiple renal cells	Finerenone	in vivo/human clinical	NLRP3 not directly assessed	[91,92]

## 7. Materials and Methods

This narrative review was developed through a targeted search of PubMed/MEDLINE, Scopus and Web of Science using combinations of terms related to “NLRP3 inflammasome”, “acute kidney injury”, “chronic kidney disease”, “pyroptosis”, “mitochondrial dysfunction”, “epigenetic regulation”, “diabetes”, “obesity”, “adipokines”, “aldosterone”, “SGLT2 inhibitors”, “GLP-1 receptor agonists” and “finerenone”. Reference lists of relevant articles were also screened to identify additional studies. Original studies, clinical investigations, and authoritative reviews in English were considered. Priority was given to recent publications, landmark mechanistic studies, and evidence relevant to renal and endocrine–metabolic disease. Studies were critically categorized according to their experimental setting patients, human tissues, animal models, or cultured cells and interpreted in relation to their mechanistic and translational relevance. Because of the narrative design, study selection was not intended to be exhaustive, and no formal risk-of-bias assessment or quantitative meta-analysis was performed.

## 8. Conclusions

The NLRP3 inflammasome is an important intersection between inflammation, mitochondrial dysfunction, pyroptosis and maladaptive repair in kidney disease, but its contribution should be interpreted in a stage- and context-dependent manner across AKI, AKD and established CKD. During AKI, NLRP3-related signaling may amplify acute inflammatory injury; during AKD, persistent signaling may contribute to incomplete recovery and maladaptive repair; and in established CKD, it may participate in chronic inflammatory-fibrotic circuits. Endocrine–metabolic disturbances, including hyperglycemia, advanced glycation end products, lipotoxicity, insulin resistance, obesity-related adipokine imbalance, and aldosterone-mineralocorticoid receptor signaling, may promote NLRP3 pathway activity. Conversely, NLRP3-mediated inflammation and pyroptosis may aggravate renal and systemic metabolic dysfunction, establishing a bidirectional pathogenic loop. Preclinical studies support a causal role for NLRP3 in selected experimental settings, whereas evidence from patients remains predominantly associative. Importantly, increased NLRP3 mRNA or protein expression alone should not be considered equivalent to inflammasome activation; functional activation is more strongly supported by downstream readouts such as ASC assembly, caspase-1 activation, mature IL-1β/IL-18 production, GSDMD cleavage, and pyroptotic cell death. NLRP3 may therefore act as a driver or amplifier of progression in some contexts, while in others its expression may primarily reflect ongoing tissue injury. Similarly, NLRP3-related signals are promising candidate biomarkers, but they have not yet been clinically validated. SGLT2 inhibitors, GLP-1 receptor agonists, and finerenone may indirectly attenuate NLRP3 signaling, although their clinical benefits cannot currently be attributed specifically to inflammasome modulation. Longitudinal human studies incorporating stage-specific phenotyping and validated target-engagement markers are required to determine the clinical relevance of NLRP3 across the AKI–AKD–CKD continuum.

## Figures and Tables

**Table 2:** Epigenetic and genetic regulators of the NLRP3 inflammasome in kidney disease. Representative microRNAs, DNA methylation changes, histone modifications, circular RNAs, and genetic variants reported to modulate NLRP3 signaling are summarized, including their direction of effect, renal or disease context, proposed mechanisms, and level of evidence. Renal evidence is distinguished from non-renal mechanistic evidence to avoid extrapolating observations from other disease systems as direct evidence of NLRP3-mediated renal pathology. The available evidence is predominantly experimental or observational, and the clinical and causal relevance of these regulatory mechanisms remains incompletely established. Symbols indicate: ↑ increased NLRP3 activation or expression, ↓ decreased NLRP3 activation or expression, and ± a context-dependent or variable effect.

Regulator	Type	Effect on NLRP3	Renal/Disease Context	Proposed Mechanism	Evidence Category	References
miR-377	miRNA	↑	Diabetic nephropathy	p38 MAPK/TXNIP/NLRP3	Animal models/cell culture	[54]
miR-155	miRNA	↑	Hypoxic/IRI tubular injury	FOXO3a/NLRP3	Animal models/cell culture	[34,55]
miR-10	miRNA	↓	Diabetic kidney disease	NLRP3 suppression	Animal models/cell culture	[33]
NLRP3 promoter methylation	DNA methylation	Context-dependent	*M. tuberculosis*-infected HEK-derived cells; T2D microvascular complications; idiopathic nephrotic syndrome	Altered transcription	Cell culture + human observational; renal and non-renal contexts	[56,57,60]
Histone acetylation	Histone modification	±	Primary macrophages; painful neuropathy (non-renal models)	NLRP3 promoter regulation	Animal models/Cell culture	[58,59]
circDENND4C	circRNA	↓	AKI	NLRP3-dependent pyroptosis	Animal models + cell culture	[66]
NLRP3/CARD8 variants	Genetic	±	CKD, lupus nephritis, diabetic nephropathy	Functional genetic variation	Human genetic association	[61,62,63,64]

## Data Availability

No new data were created or analyzed in this study. Data sharing is not applicable to this article.
